# Any old iron, man

**DOI:** 10.1113/EP092295

**Published:** 2024-10-28

**Authors:** Mike Tipton

**Affiliations:** ^1^ Extreme Environments Laboratory University of Portsmouth Portsmouth UK

The alarm was set for 04:00 h in Austria (03:00 h UK BST), but it never goes off; I have been awake with excitement and anxiety most of the night, as had the friend I am sharing the twin room with. I wonder how much a small time zone shift and one night of sleep deprivation impact on endurance performance (Lopes et al., 2022). This is seldom factored into exercise assessments. A breakfast with lots of strong coffee, an ergogenic aid (Pickering & Grgic, 2019) and cereal. Then off to bike transition at 04:45 h.

I have attempted an ‘Ironman’ (IM; 2.8 km swim, 180 km bike, 42.2 km [marathon] run) every 5 years since I gave up rugby at 43 years old; it is cheaper than private healthcare. I normally finish in 13–14 h. On the 16 June 2024, in my 66th year, it was the turn of the Austrian Ironman in Klagenfurt. Getting the excuses in early, training had not gone well due to illness and poor weather. An 8‐week respiratory viral infection made me wheezy and fatigued up to a fortnight before the event. I also had shingles and liver failure due to antibiotics (Russman et al., 2005) for a staphylococcus infection acquired from a penetrating leg injury when falling out of a tree (don't ask). I also required iron supplementation (any old iron, supplementation). Received wisdom (IM Austria course preview and hints) is that you should swim and cycle full distance at least once during training, but you only need to run about 30 km to reduce impact. I had only managed eight cycles, one over 100 km (130 km). My mantra for training as you get older is to, ‘learn to listen to your body and know when it is lying’. It is probably best to decide about a training session 10 min into it, when you have escaped the central fatigue (Newsholme & Blomstrand, 2006) induced by being stuck behind a computer before exercising.

After checking into transition, it is off to the start to change into a wetsuit. Before the event, there was some debate about whether or not the swim would be a wetsuit swim; air temperature was predicted to be 27°C, water temperature 23°C. This was a worry because you produce a lot of heat in an Ironman, with elite athletes averaging about 1100 W of heat production when cycling, and endurance athletes losing about 1 L of total body water per hour (Rogers et al., 1997). As little as 1–4% body mass loss due to dehydration can result in significant decrements in endurance performance, and dehydration has been reported to be the most common reason for medical assistance in the Hawaiian Ironman triathlon (Hiller, 1989), and dehydration and hyponatraemia (Noakes et al., 1985) become more common in races lasting longer than 7–8 h. Fear of dehydration results in some interesting compromises between fluid availability and expensive bike weight (Figure 1). The best performers tend to be able to tolerate dehydration and hyperthermia (Pugh et al., 1967); there is a limit to how much fluid the gut can absorb. Gastric emptying is reduced at intermittent exercise intensities over 75% ${\dot{V}}_{O_{2}\max}$ (Leiper et al., 2001), and drinking at a rate faster than the gut can absorb (Gisolfi et al., 1985) simply results in fluid accumulation in the stomach (Maughan & Leiper, 1999).

**FIGURE 1:**
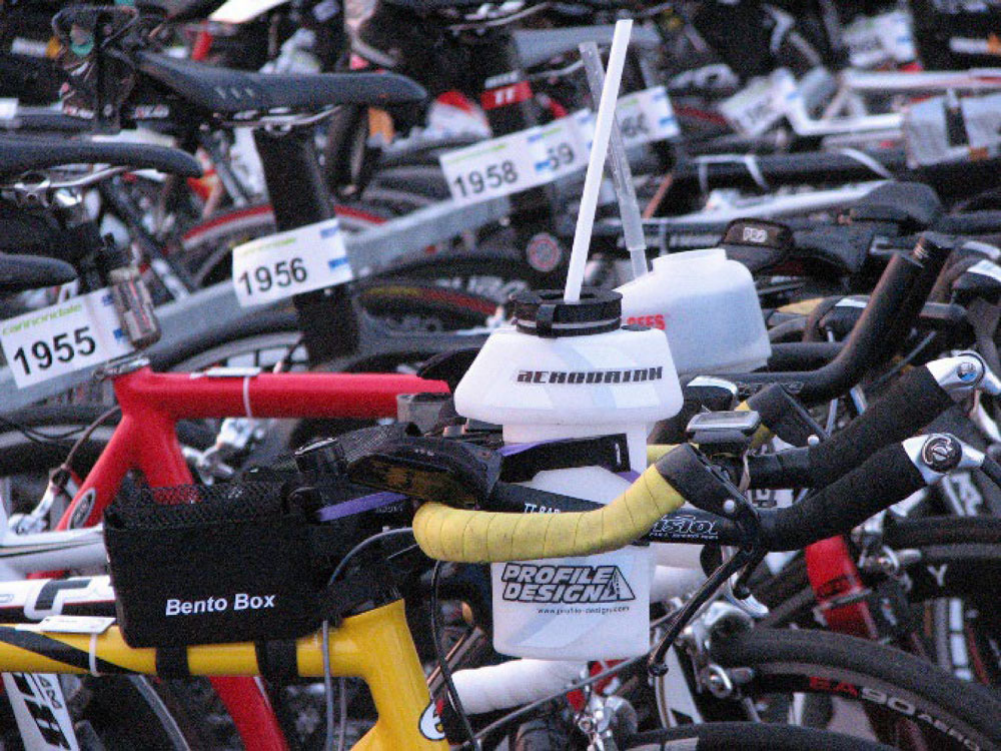
A compromise between bike weight and fluid availability.

On the day of the event, water temperature was reported to be between 18 and 20°C and a wetsuit was deemed compulsory (Saycell et al., 2018). The ‘elites’ went off at 06:30 h. The rest of us ‘age groupers’ were in pens according to our predicted swim time and were set off in groups of six at 10‐s intervals. With a predicted swim time of 70−75 min, I had to wait and then creep towards the start, which came at 07:09 h. I couldn't complain, given that the staggered, as opposed to mass, starts were a safety measure largely introduced on the basis of our work to reduce the likelihood of ‘autonomic conflict’ (Shattock & Tipton, 2012), a coincidental sympathetic and parasympathetic input to the heart that is arrhythmogenic and may help to explain why 80% of those that die in triathlons do so during the swim, despite having trained in water for many hours leading up to an event (Tipton, 2014).

Like all exercise, when you get a bit older the swim seemed hard at the start, but got easier. With the exception of loss of fitness, things take longer when you get older, longer to get fit, longer to recover from an injury or a training session, and longer to get into a steady state following the commencement of exercise (Bell et al., 1999); or perhaps it is just fitness related (George et al., 2018). Anyway, the swim went according to plan; on leaving the water there was a 500 m run to the bike transition; it is best to keep moving whilst undoing your wetsuit to avoid orthostatic blood pressure issues: some people get very light‐headed at this stage (Hansen, 2022).

After 12 min in transition, the cycle commenced. The course was two 90 km laps; the course is fast with some significant hills (Figure 2). Feeding stations were frequent, offering water, electrolytes, energy gels and, in some places, bits of banana and energy bars. But it was quite hard to feed due to the fast and then steep nature of the course (and my innate impatience).

The participants in an IM have different aims. The ‘elites’ are focussed on: *Time*, *Time*, *Time*, and worry about little else. Many of the ‘non‐elites’, including me, on: *Survival*, *Completion*, *Time*, in that order. My problems started with the realisation that I was able (for me) to go fast on the bike, hitting nearly 30 km/h in some sections and averaging 26.2 km/h on the first 90 km lap. *Time*, moved above *Survival* and *Completion*.

The second lap started well and then it happened: I ‘bonked’ (Hurley, 2024). It is pretty clear from my cycle splits (Table 1) where this happened (between 134 and 140 km). What made it worse was, that on the same section of the course that I had ‘flown’ round on Lap 1, I could barely turn the pedals on in Lap 2; this was psychologically, as well as physically, damaging.

**TABLE 1:** Comparison of cycle speed in corresponding sectors of Lap 1 and 2.

Lap 1 distance (km)	km/h	Lap 2 distance (km)	km/h	Difference in speed Lap 1 and 2 (km/h)
20.9	27.79	20.0	24.25	−3.54
30.5	28.13	30.0	26.24	−1.89
33.5	22.03	32.2	17.58	−4.45
45.0	28.66	44.0	24.10	−4.56
51.8	24.96	50.9	13.86	−11.10
70.0	22.35	69.0	12.14	−10.21
90.0	29.41	90.0	18.88	−10.53
**Mean**	**26.19**		**19.58**	

‘Bonking’ occurs when muscle glycogen is functionally depleted because the rate of utilisation exceeds the endogenous and exogenous supply. I expended about 6200 kcal during an 8‐h cycle. However, in terms of maintaining energy balance and therefore outcome, the important consideration is the substrate used to provide this energy (Jeukendrup, 2014). The energy content of an average 70 kg human comprises about 126,000 kcal from lipids and 2,000 kcal from carbohydrates; to keep going it is therefore critical to maintain carbohydrate availability. During an endurance event, blood glucose and muscle glycogen are important fuels for maintaining metabolism, and availability is determined by the balance between exercise intensity (pacing), storage and exogenous input. Blood glucose can be derived from stored liver glycogen and newly synthesised glucose, via gluconeogenesis, from substrates like glycerol, lactate and some amino acids (Webster et al., 2016).

As exercise intensity increases the percentage of carbohydrate contributing to exercise metabolism increases, and the requirement for carbohydrate is also increased (van Loon et al., 2001). Exogenous sources of carbohydrate include gels, chews and drinks, with little to choose between them (Hearris et al., 2022). Kimber et al. (2002) reported that the average carbohydrate intake during an Ironman triathlon was 1.05 g/kg BW/h (1.5 g/kg BW/h during cycling, 0.5 g/kg BW/h during running), and carbohydrate intake correlated with finishing time in male but not female athletes. There is a large literature (Jeukendrup, 2014) on the carbohydrates, and mixes of carbohydrates, it is best to ingest based on intestinal transport mechanisms and subsequent oxidation rates. This can result in some advanced nutritional strategies (Williams, 1989, 1998; Figure 3).

**FIGURE 2:**
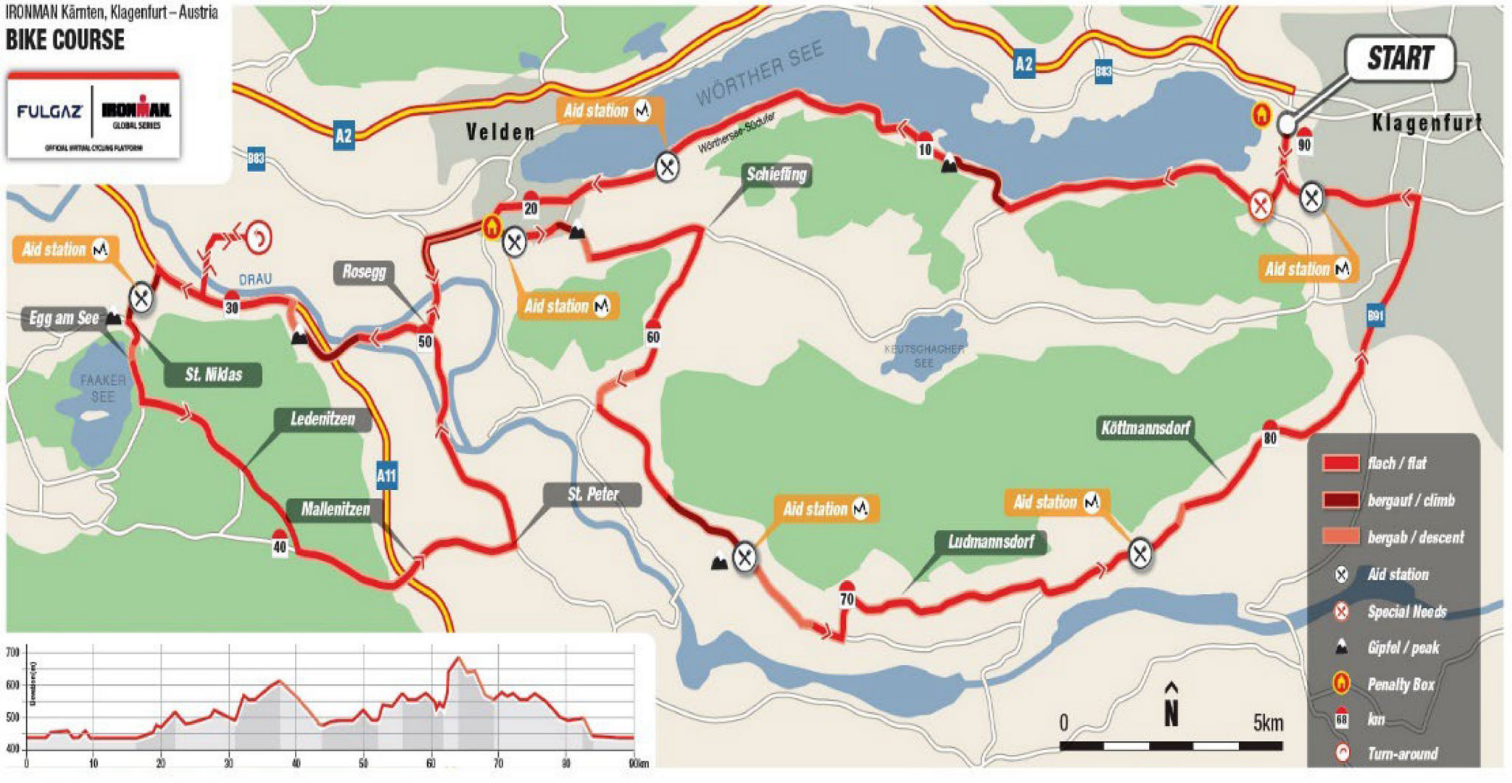
Bike course and profile – Austrian Ironman. Two laps of a 90 km course.

Other things can be done to improve carbohydrate availability. One is to increase the endogenous supply by dietary manipulations such as carbohydrate loading prior to an event; this also increases fluid storage but, as a result, increases body weight by about a kilogram. It can also cause gastrointestinal discomfort (Sedlock, 2008). Another strategy is to train with the carbohydrate source (e.g., energy gel) that will be available during an event. Apparently, such training may reduce discomfort and improve carbohydrate malabsorption and exercise‐associated gastrointestinal symptoms (Martinez et al., 2023).

For me, bonking was associated with a sensation of nausea, light‐headedness and what seemed like arrhythmia‐related compensatory beats. However, it is hard to determine the extent to which dehydration and electrolyte disturbances contributed to these symptoms. There was heavy rain in the mountains and it was warm cycling in the valleys; both cooling (cold‐induced diuresis) and heating (sweat loss) can result in ‘dehydration’. It is impossible to reverse cold‐induced diuresis with oral liquids whilst still cold, and incomplete volitional rehydration often occurs when exercising in the heat; this can be compounded by poor fluid management. Any impairment to exercising muscle blood flow, due to either heat or cold, can increase glycolytic metabolism and therefore rates of carbohydrate utilisation (Castellani & Tipton, 2015; Travers et al., 2022).

Nausea, light‐headedness and palpitations are also associated with episodes of atrial fibrillation (AFi) or atrial flutter (AFl). The relationship between high levels of physical fitness and AFi or AFl and sinus bradycardia and ventricular tachycardia is a surprise to many. The rate of atrial fibrillation, the most common serious fitness‐related arrhythmia, is 2−10 times higher in endurance athletes than controls (Turagam et al., 2015), and for every 10 years of regular endurance exercise (30 min, three times+ a week), the risk of AFi increases by about 16% and AFl by 42% (Myrstad et al., 2014). AFi and AFl can occur at rest or during exercise and have been ascribed a variety of causes including exercise‐related pressure overload on the atrium, high vagal tone and inflammatory mediators from sore muscles (Weiss & Walling, 2019). Severe hypoglycaemia is also associated with the induction of cardiac arrhythmias (Reno et al., 2013).

Fitness‐related bradycardia is a well‐known consequence of endurance training. The bradycardia is often attributed to increased parasympathetic tone, but may result from alterations in pacemaker ion channels and a reset of intrinsic heart rate (Bahrainy et al., 2016). The most potentially hazardous consequence of this bradycardia is ectopic ventricular foci breaking through the bradycardic sinus rhythm and resulting in ventricular tachycardia. A disproportionate number of ex‐professional cyclists have been reported to have ventricular tachycardia when compared to matched golfers (Baldesberger et al., 2008). With regards to symptomatic bradycardia, pacemaker insertion rates in athletic older individuals are three times those seen in matched controls (Baldesberger et al., 2008). Having said all of this, there are still many good reasons for maintaining physical activity and fitness (including agility and flexibility) into old age; not least because it is well argued that this represents the ‘human biological default condition’ (Harridge & Lazarus, 2017).

There has been an exponential increase, evident in this triathlon, in the amount of data being collected and monitored by athletes from wearable and other applicable technologies (Li et al., 2016; Rong et al., 2021). One area that requires further investigation is how athletes respond to unexpected decrements in their incoming performance data, caused by internal or external factors – a gastrointestinal issue or a rain shower. Personal observation and some published evidence (Zahrt et al., 2023), suggest that such decrements can have a negative impact on motivation, self‐esteem and mental health. Thus, it may be psychologically catastrophic for some individuals to monitor their performance plan as it disintegrates; perhaps it constrains their ability to ‘adapt and survive (*complete*)’?

There are limited options when you bonk: (a) give up or (b) stop and feed and then proceed at low intensity to minimise carbohydrate use. For a while (a) seemed like the only option. Interesting thoughts run through your fuel‐starved brain at this time; decision‐making is not at its best, tending to be driven by powerful emotive drives associated with ego, family, safety and failure. *Survival* and *Completion* swap the number one slot a lot at this time. After 5–10 min of rest and eating, (b) became more achievable and *Completion* took over as *Time* disappeared from the list (other than meeting the swim + cycle cut‐off time of 10 h 20 min). From about 160 km the cycle was mostly downhill to transition (Figure 2), so I just had to manage my nutrition and exercise intensity for the next 10–15 km and then things would become easier. By slowing significantly, my rate of carbohydrate utilisation fell and I was able to maintain it at levels where fat should be the major energy substrate (Spriet, 2014).

**FIGURE 3:**
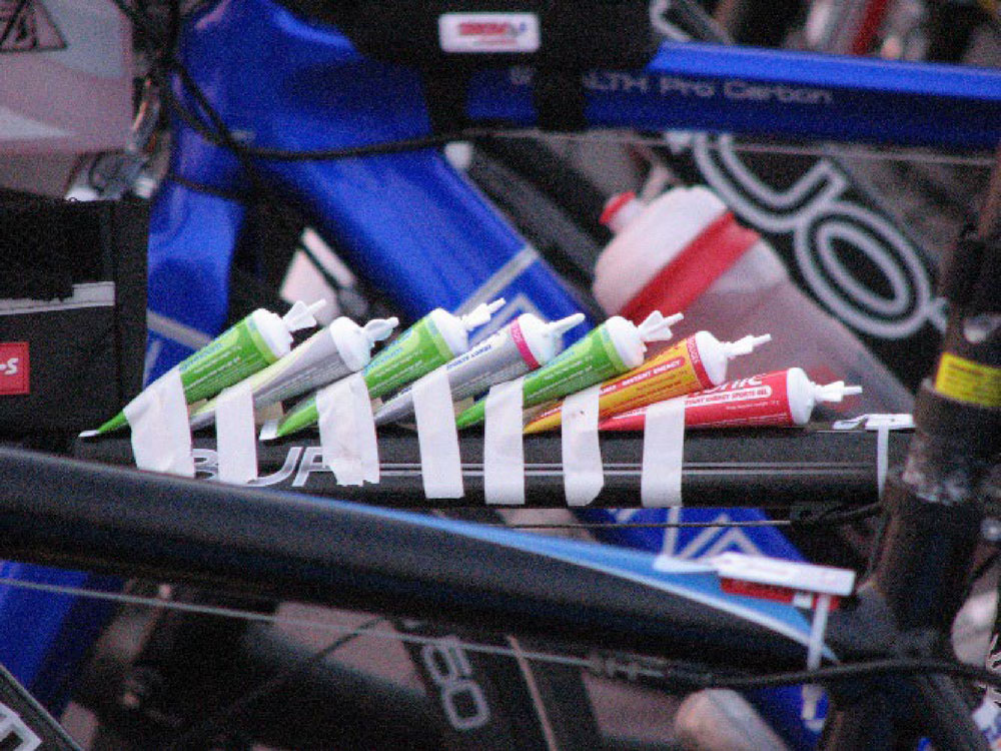
Feeding strategies can become complicated in ultra‐endurance events.

I continued this strategy in the marathon that followed, now relieved that *Survival* was probably assured. After a 7‐h Fartlek session, I crossed the line in 17 h, about 3.5 h slower than I had hoped, but a *Completion* nonetheless. A Royal National Lifeboat Institution lifeboat crew taught me a long time ago that there is a clear difference between ‘endurance ability and the ability to endure’; the former is physiology the later psychophysiology: in most ultra‐endurance events the latter applies. For the last hour of the Marathon, I periodically imagined myself running toward a pint of ‘shandy’ (Radler) (2–3% abv), my favourite post‐exercise beverage, with some scientific support (Shirreffs & Maughan, 1997; Wynne & Wilson, 2021). As luck would have it, I was offered this drink as I crossed the finish line! All's well that ends well!

In conclusion, there is a lot of integrative physiology (and psychophysiology, and pathophysiology) in an Ironman event. For non‐elite athletes in particular, each outing is something of a step into the unknown. It is a unique and, in many ways, a solitary and introspective voyage. It is also one that you can repeat into older age; my ambition to become the only person competing in my age group was dealt something of a blow by the discovery of two competitors in the 80–84‐year‐old age group. Amazing. I am due to complete my next Ironman in my 70th year. However, I am thinking of getting private healthcare.

## AUTHOR CONTRIBUTIONS

Sole author.

## CONFLICT OF INTEREST

None declared.

## FUNDING INFORMATION

None.
